# The Dual Effects of Reactive Oxygen Species on the Mandibular Alveolar Bone Formation in SOD1 Knockout Mice: Promotion or Inhibition

**DOI:** 10.1155/2021/8847140

**Published:** 2021-02-03

**Authors:** Yunyan Zhang, Yuzhi Yang, Mingxue Xu, Jingwen Zheng, Yuchan Xu, Guoqing Chen, Qiang Guo, Weidong Tian, Weihua Guo

**Affiliations:** ^1^Engineering Research Center of Oral Translational Medicine, Ministry of Education, West China Hospital of Stomatology, Sichuan University, Chengdu, China; ^2^National Engineering Laboratory for Oral Regenerative Medicine, West China Hospital of Stomatology, Sichuan University, Chengdu, China; ^3^State Key Laboratory of Oral Diseases, National Clinical Research Center for Oral Diseases, West China Hospital of Stomatology, Sichuan University, Chengdu, China; ^4^Department of Pediatric Dentistry, West China Hospital of Stomatology, Sichuan University, Chengdu, China; ^5^Department of Oral and Maxillofacial Surgery, West China Hospital of Stomatology, Sichuan University, Chengdu, China

## Abstract

The status of reactive oxygen species (ROS) correlates closely with the normal development of the oral and maxillofacial tissues. Oxidative stress caused by ROS accumulation not only affects the development of enamel and dentin but also causes pathological changes in periodontal tissues (periodontal ligament and alveolar bone) that surround the root of the tooth. Although previous studies have shown that ROS accumulation plays a pathologic role in some oral and maxillofacial tissues, the effects of ROS on alveolar bone development remain unclear. In this study, we focused on mandibular alveolar bone development of mice deficient in superoxide dismutase1 (SOD1). Analyses were performed using microcomputerized tomography (micro-CT), TRAP staining, immunohistochemical (IHC) staining, and enzyme-linked immunosorbent assay (ELISA). We found for the first time that slightly higher ROS in mandibular alveolar bone of SOD1(-/-) mice at early ages (2-4 months) caused a distinct enlargement in bone size and increased bone volume fraction (BV/TV), trabecular thickness (Tb.Th), and expression of alkaline phosphatase (ALP), Runt-related transcription factor 2 (Runx2), and osteopontin (OPN). With ROS accumulation to oxidative stress level, increased trabecular bone separation (Tb.Sp) and decreased expression of ALP, Runx2, and OPN were found in SOD1(-/-) mice at 6 months. Additionally, dosing with N-acetylcysteine (NAC) effectively mitigated bone loss and normalized expression of ALP, Runx2, and OPN. These results indicate that redox imbalance caused by SOD1 deficiency has dual effects (promotion or inhibition) on mandibular alveolar bone development, which is closely related to the concentration of ROS and the stage of growth. We present a valuable model here for investigating the effects of ROS on mandibular alveolar bone formation and highlight important roles of ROS in regulating tissue development and pathological states, illustrating the complexity of the redox signal.

## 1. Introduction

Reactive oxygen species (ROS), a natural product of cellular aerobic metabolism, are chemically reactive molecules such as superoxide (O_2_^·-^), hydrogen peroxide (H_2_O_2_), and the hydroxyl radical (HO^·^). In earlier studies, oxidative stress caused by ROS accumulation has been linked to multisystem diseases associated with inflammation and aging. Recent studies have found that ROS play several important roles in cancer therapy, immunity, autophagy, and, especially, signal transduction [1–3]. In the low range, ROS perform complex signaling functions as a signaling molecule that regulates cell growth and differentiation [4]. However, high-concentration ROS may also contribute to oxidative denaturation of intracellular proteins and lipids, DNA damage, alteration of cell signaling pathways, and cell apoptosis and ultimately lead to the body's aging [5]. To regulate ROS concentrations, the body has an antioxidant enzyme system and antioxidant substances that eliminate ROS to achieve balance. It is well established that SODs are the first line of defense against ROS by catalyzing the disproportionation of superoxide radicals into hydrogen peroxide and molecular oxygen. There are three known SOD subtypes classified mainly by location in mammals: SOD1 present in cytoplasm and mitochondrial intermembrane, SOD2 localized at the mitochondrial matrix, and SOD3 being secreted to the extracellular space [6]. Most abundant and expressed in the majority of tissues, SOD1 has a surprisingly high cellular concentration [7] (estimated to be 10–40 *μ*M). Moreover, unlike SOD2(-/-) mice, homozygous mutations in the SOD1 gene are not lethal; still, SOD1(-/-) mice exhibit subtle defects, including Amyotrophic Lateral Sclerosis (ALS), retinal dysfunction, and muscle atrophy [8–11]. Therefore, SOD1-deficient mice are a verified animal model for studying the effects of ROS aggregation on tissue/organ development and disease occurrence and treatment.

Models established by methods such as a high-fat diet [12], stimulation of cells with sodium fluoride [13], and H_2_O_2_ dosage [14, 15] showed that oxidative stress can cause alterations in oral and maxillofacial tissues, causing premature senescence of dental pulp cells [15], apoptosis of enamel cells, interference of tooth mineralization [12–14], abnormal tooth development, periodontal diseases [15], and oral cancer [16]. Previous studies have demonstrated that SOD1 knockout mice showed bone disturbances. A study of the femur found that in SOD1-deficient subjects, ROS accumulation caused reduced bone formation, absorption, and low bone remodeling activity, which resulted in accelerated bone loss and increased bone fragility [17]. Another study demonstrated that femurs of SOD1 knockout mice were weaker than those of wild-type mice during puberty skeletal growth [18]. Furthermore, ROS have been implicated in bone metabolism by mediating osteoclastic resorption [19] and osteoblastic differentiation [20]. Excessive ROS may cause apoptosis of osteoblasts and consequently bone metabolic disorders [21].

The alveolar bone and teeth develop closely and together form the dentoalveolar complex. The alveolar bone anchors the teeth. Disruptions in alveolar bone development and maintenance can result in pathological changes such as bone loss or osteoporosis, which may lead to mobile teeth and maxillofacial bone diseases, affecting physiological function as well as aesthetics. Excessive ROS accumulation has an adverse impact on the development of long bones and some oral and maxillofacial tissues. However, research on the effects of ROS on the development of mandibular alveolar bone is absent. Hence, we hypothesized that ROS aggregation impacts mandibular alveolar bone formation. Specifically, we established a SOD1 knockout mouse model and demonstrated that ROS aggregation could have dual effects on mandible formation: promotion or pathological effect, which depended on ROS levels and development stage. Furthermore, treatment with the antioxidant NAC effectively improved bone loss and increased expression of osteogenesis protein markers.

## 2. Materials and Methods

All animal experiments were conducted in accordance with the principles and procedures of the National Institutes of Health (NIH) Guide for the Care and Use of Laboratory Animals and approved by the Ethics Committee of the State Key Laboratory of Oral Diseases, West China Hospital of Stomatology (Chengdu, Sichuan) (license number WCHSIRB-D-2016-195).

### 2.1. Experimental Animals, Genotyping, and Grouping

SOD1(-/-) mice and corresponding SOD1(+/-) and SOD1(+/+) controls were generated from the intercross between congenic B6 <Sod1+/->; 129S-Sod1tm1Leb/J, purchased from Shanghai Southern Model Biotechnology Co., Ltd. All mice were housed in the pathogen-free facility at Sichuan University Experimental Animal Center and given SPF animal feed and drinking water. The photoperiod was carried out according to the law of 12 : 12 circadian rhythm. The illumination intensity was 130-325 lux, the room temperature was 21 ± 3°C, the relative humidity was 40-60%, the noise was 25-40 dB, and the air exchange times were 10-15 times/hour. Due to female infertility of SOD1(-/-) mice, SOD1(+/-) mice were selected for breeding and colony maintenance. SOD1 (+/-) knockout mice older than 8 weeks were selected as breeders, 1♂×2♀ per cage. After mating, as shown in Figure S1, SOD1(-/-) (homozygous), SOD1(+/-) (heterozygous), and SOD1 (+/+) (wild type) were obtained. The genotyping of each mouse was assessed by quantitative PCR of DNA isolated from tail biopsy samples, as described on the Jackson Laboratory web site. Animals were divided in four groups: (1) normal diet (WT) group, (2) normal diet (SOD1-/-) group, (3) NAC-supplemented diet (SOD1-/-) group, and (4) NAC-supplemented diet (WT) group. The antioxidant drug N-acetylcysteine (NAC) was added to the drinking water at a concentration of 1 mg/mL. The NAC concentration used in this study was similar to the concentration used in previous studies [22, 23].

### 2.2. Enzyme-Linked Immunosorbent Assay (ELISA)

Mouse serum samples were collected. After pretreating collected containers with sodium heparin, we performed tail-cut blood collection of male mice at 2 months, 4 months, and 6 months. Samples were centrifuged at 3000 g for 25 minutes at 4°C to collect the upper serum. To determine the amount of lipid peroxidation in the serum, the MDA levels were analyzed spectrophotometrically using the modified thiobarbituric acid-reactive substance method as described by Zhou et al. [24]. The results were expressed as nmol/mL. The serum level of SOD, GSH-PX, and H_2_O_2_ were detected via specific kits (Jiancheng, Nanjing, China) following the manufacturer's instructions.

### 2.3. Mandible Morphometric Analyses by Microcomputerized Tomography (Micro-CT)

Scans for volume, density, and bone mass of mandible alveolar bone were performed with a microtomograph imaging system (*μ*CT 50, Scanco Medical AG, Brüttisellen, Switzerland). The scanning parameters were X-ray bulb voltage 90 kV, current 200 *μ*A, resolution 9 *μ*m, and exposure time 500 ms. The images were processed by three-dimensional reconstruction software (*μ*CT Evaluation Program v6.0, Scanco Medical) and analyzed for determination of bone mass and density.

Bone mass analysis was performed on the alveolar bone of the first molar root bifurcation. The VOI (volume of interest) of the analysis region was selected as a cube with a length of 270 *μ*m below the alveolar crest of the left molar mandibular root bifurcation. The middle 30 layers were selected for trabecular bone parameter analysis, including bone mineral density (BMD), bone volume fraction (bone volume per total volume, BV/TV) (%), trabecular thickness (Tb.Th), trabecular separation (Tb.Sp), and trabecular bone number (the mean trabecular number, Tb.N). Each sample was analyzed three times to calculate the average value.

### 2.4. Histology and TRAP Staining

Mandibles were removed, fixed in 4% paraformaldehyde overnight at 4°C, and decalcified in 10% EDTA for 1 month at 37°C. Decalcified mandibles were dehydrated and embedded in paraffin and cut at 5 *μ*m per slice on a rotary microtome. The sections were stained with alkaline tartrate-resistant acid phosphatase (TRAP) activity and immunohistochemically as described below. The number of osteoclasts per area, TRAP (+) cells/mm^2^, was identified by the TRAP staining. All cells with 2 or more nuclei and close to trabeculae were considered osteoclastic cells. After applying TRAP solution (0.1 macetic acid buffer solution; naphthol AS-MX phosphate; fast red violet LB salt; N,N-dimethylformamide; Sigma-Aldrich Inc.), the sections were incubated at 37°C for 30 min. A counterstaining with methyl green staining for 3 minutes was performed. The number of osteoclasts was measured using Image-Pro Plus 6.0 software (Rockville, MD). Three independent operators observed the sections and did the counting under the microscope.

### 2.5. Immunohistochemical Staining

Briefly, dewaxed and rehydrated paraffin-embedded sections were incubated with 3% (*w*/*v*) hydrogen peroxide for 10 minutes to block endogenous peroxidase activity and then washed twice in PBS. Antigen recovery was performed with pepsin antigen repair solution at room temperature for 10 minutes and then washed with PBS for 10 minutes. Blocking was completed with 5% (*w*/*v*) goat serum for 2 hours at room temperature. The slices were then incubated with the primary antibodies overnight at 4°C. After washing with PBS, sections were incubated with secondary antibody (Sigma, St. Louis, MO, USA) for 30 minutes. After washing, brown pigmentation was likewise produced using 3,3-diaminobenzidine (DAB). Finally, the stained sections were counterstained with hematoxylin. Images were acquired with a Leica microscope (Leica DM4000B, Solms, Germany) equipped with Leica software. Primary antibody included antibodies against Runx2 (ab102711; Abcam), alkaline phosphatase (ALP) (ab108337; Abcam), osteopontin (OPN) (ab8448; Abcam), and 4-hydroxynonenal (4-HNE) (ab46545; Abcam) at a dilution of 1 : 200 according to the manufacturers' protocol. Approximately 6–8 sections were obtained from each sample of the same region. We used the Image-Pro Plus 6.0 software to compare the mean density (IOD/area) results with the same parameter settings for each sample.

### 2.6. Statistical Analysis

The data were analyzed using GraphPad Prism software (4.0) (San Diego, CA). Statistical analysis of data was performed using analysis of ANOVA and Student's *t*-test, followed by the Student–Newman–Keuls post hoc test for multiple group comparisons. Results are presented as mean ± SD. Differences with a *P* < 0.05 are considered statistically significant. All in vitro experiments were repeated at least three times.

## 3. Results

### 3.1. Mice Deficient in SOD1 (SOD1(-/-) Mice) Displayed Developmental Delays and Signs of Overall Oxidative Stress

**Figure 1:**
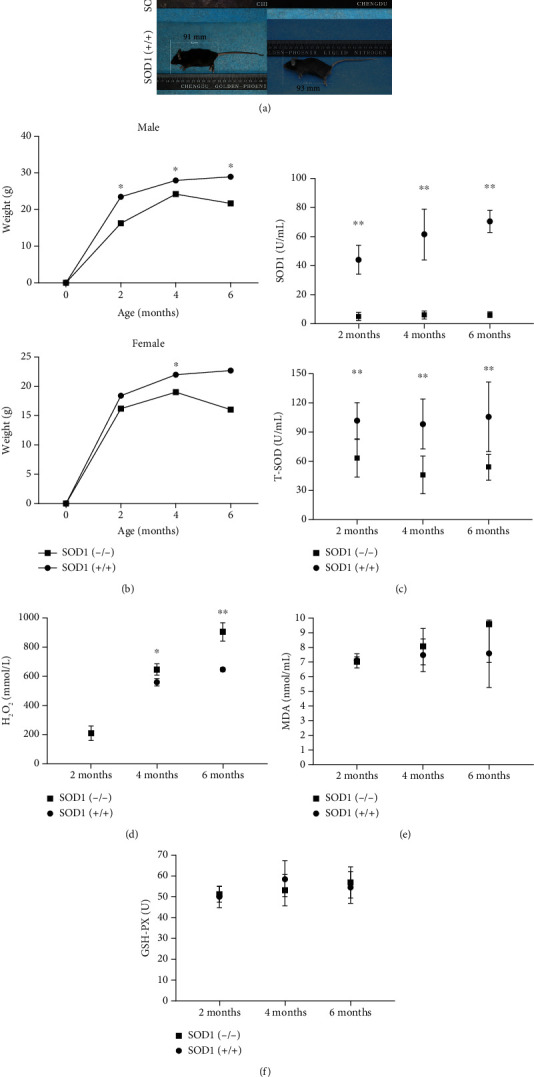
Analysis of oxidative stress state in SOD1(-/-) mice and wild-type mice. (a) SOD1(-/-) mice have smaller body size than age-matched wild-type mice (3 months). (b) Body weight measurement of SOD1(-/-) mice and wild-type mice at different time periods. (c) Serum ELISA shows successful deleting of SOD1 and total-SOD reduction in SOD1(-/-) male mice. (d, e) Serum ELISA of H_2_O_2_ and oxidative damage product (MDA) show obvious ROS accumulation in SOD1(-/-) male mice. (f) Serum ELISA of GSH-PX reductase shows no difference between SOD1(-/-) male mice and wild-type mice. Date are shown as mean ± SD (*n* = 5). ^∗^*P* < 0.05; ^∗∗^*P* < 0.01.

Establishment of the SOD1 knockout mouse model was confirmed by successful deletion of SOD1 and total-SOD reduction in serum using ELISA (Figure 1(c)). At the same time, we measured the level of GSH-PX reductase in mice and found no obvious compensation with the decrease in SOD reductase (Figure 1(f)), which meant that the defense ability of the enzyme antioxidant system against oxygen free radicals was dysfunctional. SOD1(-/-) mice exhibited lower weights and smaller body sizes compared to age-matched wild-type mice (Figures 1(a) and 1(b)), indicating that lack of SOD1 affected the growth and development of the whole body. SOD1(-/-) mice at 4-6 months exhibited a slight decrease in body weight, which might be related to mouse skeletal muscle atrophy [25].What is more, by detecting hydrogen peroxide and oxidative damage product MDA, we found that the level of serum H_2_O_2_ and MDA in the SOD1(-/-) mice were similar to those in the wild-type mice at 2 months of age (*P* > 0.05) (Figures 1(d) and 1(e)). As mice aged, the levels of H_2_O_2_ and MDA in the SOD1(-/-) mice increased. The differences of serum H_2_O_2_ and MDA levels in SOD1(-/-) mice compared to controls were obvious at 4 months and 6 months (*P* < 0.05) (Figures 1(d) and 1(e)). In conclusion, the accumulation of ROS due to the deficiency of SOD1 gradually appeared with age, ultimately leading to oxidative stress.

### 3.2. Age-Related ROS Accumulation Caused Dual Effects on Mandible Development

**Figure 2:**
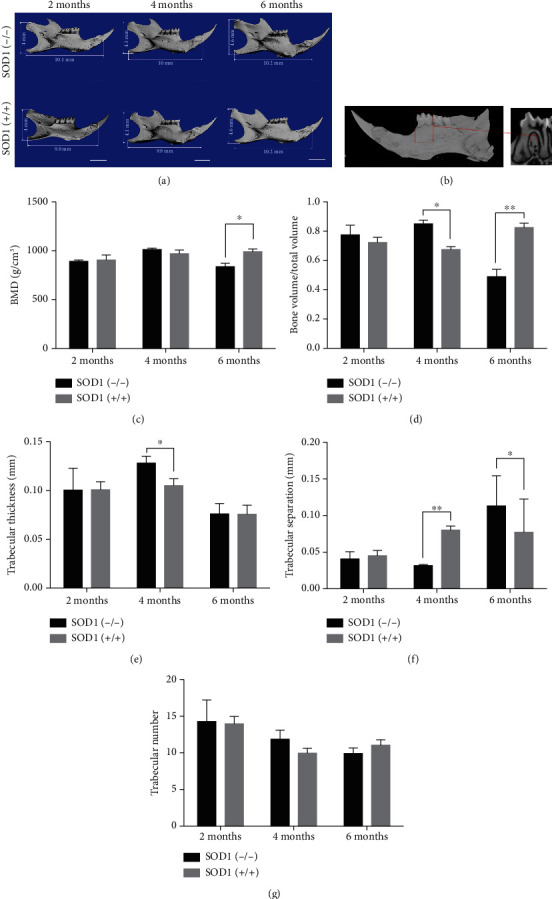
Morphological characteristics and micro-CT of the mandibles in SOD1(-/-) male mice and wild-type male mice. (a) Microcomputed tomography reconstruction of mandibles at 2 months,4 months, and 6 months. (b) Quantitative analysis of alveolar bone under the alveolar crest of the mandibular first molar root bifurcation area. (c–g) Bone mineral density (BMD), bone volume fraction (BV/TV), trabecular thickness (Tb.Th), trabecular bone separation (Tb.Sp), and trabecular bone number (Tb.N) of alveolar bone in first molar root bifurcation area. Data are shown as mean ± SD (*n* = 5). ^∗^*P* < 0.05; ^∗∗^*P* < 0.01. Scale bars (a) 2 mm.

Three-dimensional scanning of SOD1(-/-) mice and the wild-type mandibles and analysis of the bone mass of alveolar bones were performed at 2 months, 4 months, and 6 months of the Figure 2. Previous studies reported that SOD1 gene knockout affected ovarian function, leading to abnormal secretion of follicle stimulating hormone (FSH), luteinizing hormone (LH), and progesterone (P) [26]. At the same time, these estrogens could affect the growth and development of the mandible [27], so male mice were used when analyzing the mandibular alveolar bone. Interestingly, the size of the 2-month-aged SOD1(-/-) mice's mandibular bone was larger than its counterpart in the wild-type mice. From 2 to 6 months of age, the mandible size of SOD1(-/-) mice did not change much while the wild-type mice showed progressive growth of mandibles. At 6 months of age, the mandible sizes of the SOD1(-/-) mice and the wild-type mice were basically similar. As shown in Figure 2, we did quantitative analysis of alveolar bone under the alveolar crest of the mandibular first molar root bifurcation area. At 2 months of age, bone mineral density (BMD), bone volume fraction (BV/TV), trabecular thickness (Tb.Th), trabecular bone separation (Tb.Sp), and trabecular bone number (Tb.N) in the SOD1(-/-) mice were not statistically significantly different compared with the wild-type mice. At 4 months of age, compared with the wild-type mice, the bone volume fraction and trabecular thickness of the male SOD1(-/-) mice increased (Figures 2(d) and 2(e)), and the trabecular bone separation decreased (*P* < 0.05) (Figure 2(f)). At 6 months of age, the results were opposite. Compared with the wild-type mice, the bone mineral density and bone volume fraction of the male SOD1(-/-) mice were reduced (Figure 2(d)), and the trabecular bone separation was increased (*P* < 0.05) (Figure 2(f)).

### 3.3. Low Osteoclast Amount and Mutative Osteogenic Associated Protein Expression in an Age-Dependent Manner Were Observed in SOD1(-/-) Mice

**Figure 3:**
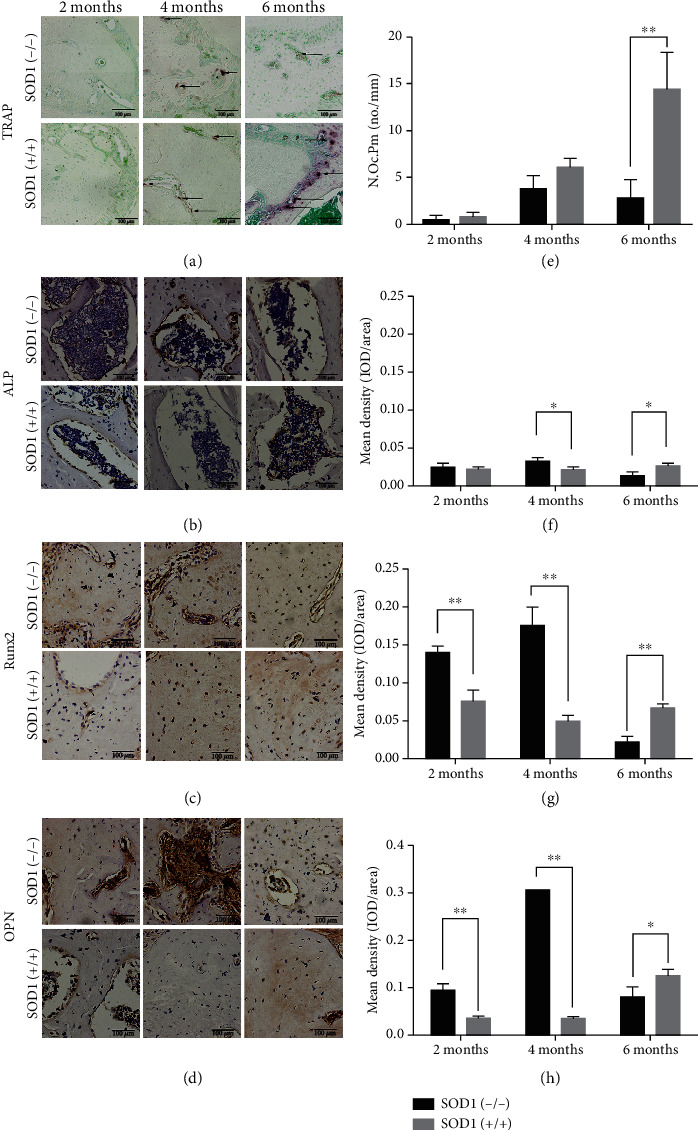
Bone metabolism of the mandible in SOD1(-/-) male mice and wild-type male mice. (a) TRAP staining shows lower number of osteoclasts in alveolar bone under the alveolar crest of the mandibular first molar root bifurcation area of SOD1(-/-) mice. Arrows indicate active osteoclasts. (b) Immunohistochemistry of ALP in the same place. (c) Immunohistochemistry of Runx2 in the same place. (d) Immunohistochemistry of OPN in the same place. (e–h) Quantitative analysis of (a–d), respectively. Data are shown as mean ± SD (*n* = 5). ^∗^*P* < 0.05; ^∗∗^*P* < 0.01. Scale bars (a–d) 100 *μ*m.

In healthy bone tissue, a delicate homeostasis is maintained between the action of the osteoclasts in bone resorption and the action of osteoblasts in bone formation. We observed the number of osteoclasts through TRAP staining in the alveolar crest of the mandibular first molar root bifurcation area (Figure 3(a)). In both wild-type mice and SOD1(-/-) mice, there were almost no TRAP-positive osteoclasts in the alveolar bone at 2 months of age. With increasing age, the number of osteoclasts increased in the wild-type mice. In the SOD1(-/-) mice, the counts of osteoclasts increased, yet at a lower amount compared to wild-type mice. At 6 months of age, the number of TRAP-positive osteoclasts in SOD1(-/-) mice was significantly lower than that in the wild-type mice (*P* < 0.01), indicating that SOD1 deficiency impaired bone resorption. At the same time, we also performed immunohistochemistry of the transcription factor Runx2 and osteogenesis markers OPN and ALP in mandibles (Figure 3). In earlier ages (2 months), the expression levels of Runx2 and OPN in the SOD1(-/-) mice were significantly higher than those in the wild-type mice (Figures 3(g) and 3(h)). At 4 months, the expression levels of Runx2, OPN, and ALP remained higher compared to the wild-type mice. Notably, a sharp reduction of Runx2 and OPN could be found at the age of 6 months in the SOD1(-/-) mice (Figures 3(c) and 3(d)), indicating that the ability of alveolar bone formation in SOD1 knockout mice also increased at first and then decreased over time. Generally, 4-HNE, a biofilm lipid peroxidation product, can reflect the degree of oxidative damage in the alveolar bone region [28]. Immunohistochemistry of 4-HNE in alveolar bone showed that the oxidative stress of the jaw had been becoming more and more obvious with age in SOD1(-/-) mice (as shown in Figure S2).

### 3.4. Antioxidant NAC Application Reversed Oxidative Stress and Caused Low ROS Concentration in SOD1(-/-) Mice

**Figure 4:**
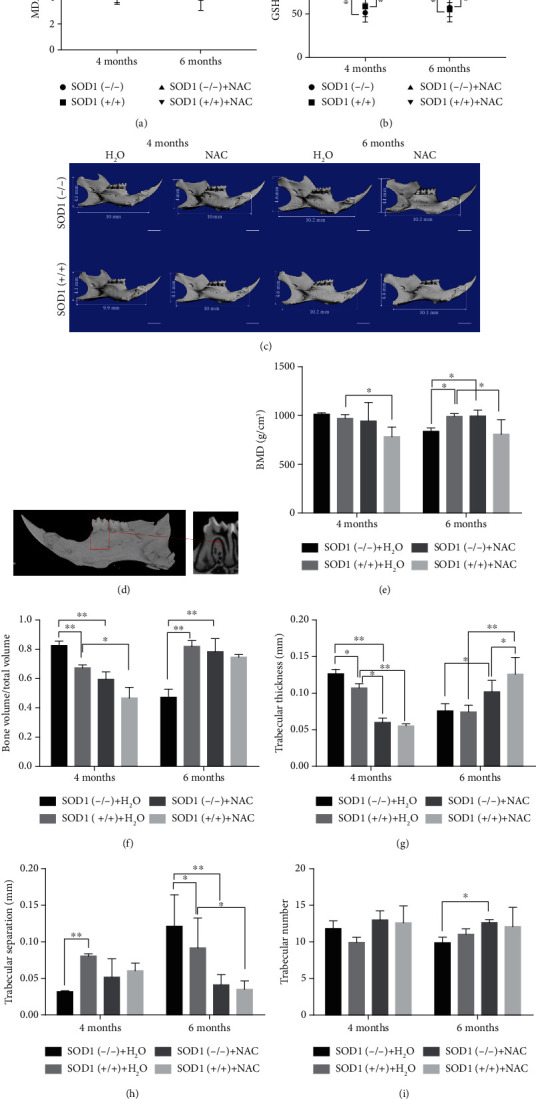
Antioxidant NAC application reverse oxidative stress and affect mandible formation. (a) ELISA shows lower MDA level in serum after adding antioxidant NAC in the diet of mice. (b) ELISA shows higher GSH-PX level in serum after adding antioxidant NAC in the diet of mice. (c) Microcomputed tomography reconstruction of mandibles of SOD1(-/-) male mice after feeding with NAC. (d) Quantitative analysis of alveolar bone under the alveolar crest of the mandibular first molar root bifurcation area. (e–i) Bone mineral density (BMD), bone volume fraction (BV/TV), trabecular thickness (Tb.Th), trabecular bone separation (Tb.Sp), and trabecular bone number (Tb.N) of alveolar bone in first molar root bifurcation area. Data are shown as mean ± SD (*n* = 5). ^∗^*P* < 0.05; ^∗∗^*P* < 0.01. Scale bars (c) 2 mm.

One of the most commonly used antioxidants is N-acetylcysteine (NAC), which is the precursor of reduced glutathione (GSH) in cells [29]. Increasing the content of NAC can promote the synthesis of GSH-PX and help remove the excess active oxygen free radicals [30, 31]. After adding antioxidant NAC to the diet of mice, the MDA levels in serum and 4-HNE levels in alveolar bone of the SOD1(-/-) mice showed significant decline compared with those in the normal feeding group (*P* < 0.01) (Figure 4(a) and Figure S3). More importantly, under normal feeding conditions, SOD1(-/-) mice had higher serum MDA levels and 4-HNE levels in alveolar bone than wild-type mice, but the difference was eliminated after adding NAC. Interestingly, the serum MDA content and 4-HNE levels in alveolar bone of the SOD1(-/-) mice feeding with NAC were even lower than the wild-type mice on normal diet and did not change significantly with age and remained below physiological levels (Figure 4(a) and Figure S3). Moreover, after adding antioxidant NAC in the diet of mice, serum GSH-PX levels in the SOD1(-/-) mice and the wild-type mice showed no significant difference between each other (*P* > 0.05), whereas, compared with normal diet groups, both SOD1(-/-) mice and wild-type mice exhibited higher levels of GSH-PX after adding NAC (*P* < 0.01) (Figure 4(b)).

### 3.5. Effects of NAC-Induced Decrease in ROS Concentration on Jaw Formation and Bone Metabolism of SOD1(-/-) Mice

**Figure 5:**
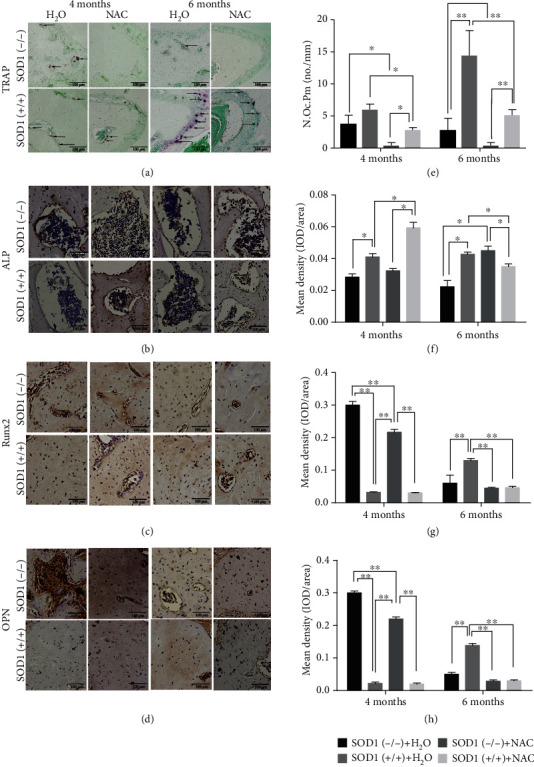
Antioxidant NAC application affect bone metabolism. (a) TRAP staining shows lower number of osteoclasts after adding NAC in a diet of mice. Arrows indicate active osteoclasts. (b) Immunohistochemistry of Runx2. (c) Immunohistochemistry of OPN. (d) Immunohistochemistry of ALP. (e–h) Quantitative analysis of (a–d), respectively. Data are shown as mean ± SD (*n* = 5). ^∗^*P* < 0.05; ^∗∗^*P* < 0.01. Scale bars (a–d) 100 *μ*m.

Micro-CT analysis showed that the mandibular size of the SOD1(-/-) mice after 2 months of NAC feeding (4 months old) was larger than that of the normal feeding group (Figure 4(c)). After 4 months (6 months old), we found that BMD, BV/TV, Tb.Th, and Tb.N increased in the SOD1(-/-) NAC group, compared with the SOD1(-/-) normal feeding group. No significant differences were found in BMD and bone mass (BV/TV) between the normal wild-type group and the SOD1(-/-) NAC group (*P* > 0.05) (Figures 4(e) and 4(f)). It is noteworthy that BMD, BV/TV, and Tb.Th decreased after adding NAC in the diet in the wild-type mice at 4 months old (*P* < 0.05) (Figures 4(e) and 4(f)). The number of osteoclasts in both the SOD1(-/-) group and the wild-type group was significantly reduced after adding NAC in the diet (*P* < 0.05) (Figure 5(a)). Although ROS aggregation had been reversed and ROS concentration had shrunk, the alveolar bone remodeling activity was maintained at a low level. As for the bone formation, comparing the wild-type NAC group with the wild-type normal feeding group, we found that Runx2 and OPN did not change significantly at 2 months (4 months old), while decreases were observed at 4 months after the dosing (6 months old) (Figures 5(c) and 5(d)), indicating that reduction of ROS concentration to a certain extent would lead to weaker osteoblast activity. At 6 months old, SOD1(-/-) mice exhibited obvious bone loss. We found that the use of NAC significantly increased the expression of ALP and Runx2 (Figures 5(f) and 5(g)).

## 4. Discussion

Since Denham Harman proposed the free radical doctrine in 1956, many degenerative diseases such as heart disease, Alzheimer's disease, Parkinson's disease, and tumors have been shown to be linked to excessive free radicals [32–34]. Through signaling, regulating stem cell fate, and immunity, ROS play an important role in maintaining homeostasis and promoting normal development [35], but the specific role in various tissues and organs has not yet been proven definitively.

Previous studies have shown that ROS accumulation caused by SOD1 dysregulation can lead to increased bone mass reduction, skeletal fragility, and low-turnover osteoporosis in long bones [18, 36]. In the current study, we demonstrated for the first time that increased ROS levels caused by SOD1 deficiency resulted in enlargement of mandible size and increased bone mass at early age (2-4 months). In contrast, mandibles of SOD1(-/-) mice at older age (6 months) exhibited a significant reduction in bone mass. These data suggest that ROS aggregation has dual effects on mandibular alveolar bone: promotion or pathogenic effect, which depends on ROS levels and stages of development. Notably, the phenotypes of long bones and mandibles were not consistent despite the same age of SOD1(-/-) mice (4 months). Nojiri et al. [18] found decreases in total bone density, BV/TV, Tb.N, and Tb.Th and an increase in trabecular separation in the femur of SOD1(-/-) mice. Our results in mandibular alveolar bones demonstrated the opposite, suggesting that different types of bones have different responses and tolerances to ROS changes. Mandibular alveolar bones and long bones have different developmental origins and responses. Mandibles originate from the neural crest of the ectoderm, while mesenchymal cells of the mesoderm develop into long bones. Previous studies have found that bone marrow mesenchymal stem cells derived from rat mandibles had significantly higher proliferation capacity and mineralization ability in vitro than bone marrow mesenchymal stem cells derived from long bones [37]. Moreover, in some diseases, such as osteoporosis and hyperthyroidism, the degree of involvement in long bones is significantly higher than that in the jaws [38].

In the early stages of mandible development (2 months), the cranial and maxillofacial regions of C57 mice generally grow most rapidly (close to 80% of the growth is completed) [39]. Although overall ROS levels are not elevated and oxidative stress is low, cytoplasmic and mitochondrial ROS may be slightly elevated [40]. Therefore, enlargement of mandible size may result from H_2_O_2_ slowly accumulating in the cell, exerting its role as a signal molecule, and promoting the proliferation and differentiation of bone marrow stem cells in the initial stage of SOD1 deficiency [41]. With the increase of age (4 months), we found higher expression of osteogenesis-related proteins (Runx2, ALP, and OPN) and fewer osteoclasts in mandibles of SOD1(-/-) mice (Figure 3). At the same time, we also found that SOD1(-/-) mice mandibles had higher levels of 4-HNE than those of the wild-type mice. Interestingly, previous studies showed that 4-HNE can act as a signaling molecule to promote cell proliferation and affect transcription at a certain concentration [42]. Increased expression of ALP, OPN, and Runx2 may be associated with mild elevated H_2_O_2_ and 4-HNE levels in mandibular alveolar bones of the SOD1(-/-) mice. These results indicated that the increase of ROS may promote the mineralization of the bone matrix and the deposition of calcium when the development of the mandibular alveolar bone is basically stable. At the same time, elevated ROS level may reduce bone resorption, thereby increasing the bone mass and density of the mandibular alveolar bone.

When ROS continued to accumulate, at 6 months of age, the mandibular alveolar bone showed obvious oxidative stress, decreased BMD and bone mass, and decreased ALP, OPN, and Runx2 expressions (Figures 2 and 3). Despite the obvious bone loss, our results showed that the number of TRAP-positive osteoclasts reduced, which support the idea that accumulated ROS may have resulted in the suppression of osteoclastogenesis and low-turnover bone loss [18]. Another possibility is increased ROS-induced ROS release (RIRR), which means that elevated ROS function through diminishing the efficacy of antioxidant defenses and inducing the activation of transcription factors of ROS-producing enzymes to increase ROS production [43–45]. The rapid expansion of ROS may cause severe oxidative stress in jaw bones, inducing apoptosis and autophagy [46, 47], which damages the function and reduces the number of osteoblasts and osteoclasts, thereby destroying bone homeostasis and contributing to bone diseases.

Considering the concentration-dependent effect of ROS [48], controlling ROS in the appropriate range is a potential treatment. Given that SOD reductase is easily metabolized after entering the body for the poor tissue affinity and instability [49], we use the antioxidant NAC, the precursor of reduced glutathione (GSH) in the cell, to help clear excess reactive oxygen radicals. In our study, we demonstrated that N-acetylcysteine could reverse the pathogenic impact of ROS aggregation on the mandibular alveolar bone: improving bone formation and increasing bone mass. The increase in the content of NAC can promote the synthesis of GSH (Figure 4) and help remove excess reactive oxygen species accumulated in the body [50]. Balszuweit et al. [32] also reported that NAC protected against LPS-induced inflammatory bone loss via reducing ROS formation in macrophages and osteoclasts. Mandibular alveolar bone oxidative stress level in SOD1(-/-) mice, displayed by levels of 4-HNE and MDA, was effectively alleviated, even lower than normal condition after the addition of antioxidant NAC (Figure 4), which suggested it also could be used as a model to study the effect of ROS reduction on mandibular alveolar formation. Our results showed that SOD1(-/-) mice have significantly improved BMD, BV/TV, Tb.Th, and Tb.N of mandibular alveolar bone after taking NAC for 4 months (6 months old), indicating that superoxide-induced bone loss could be improved by antioxidant treatment. Additionally, NAC treatment also produced increased osteogenesis-related protein expression (ALP, Runx2, and OPN) and decreased number of osteoclasts. However, in the wild-type mice at 6 months, the use of NAC led to reduced BMD, bone mass, and expression of ALP, Runx2, and OPN. These results imply that NAC might protect against bone loss via reducing ROS formation in macrophages and osteoclasts [51]. The reduction of ROS caused by antioxidants, even lower than normal physiological concentration, may inhibit osteogenesis. Previous studies showed that excessively low levels of ROS can also cause pathological changes by disturbing the physiological role of oxidants in proliferation, vasodilation, and host defense and may lead to cancer development [52]. Therefore, excessively low ROS concentration is not conducive to mandible development. Together with our results, these reports suggest that NAC could represent a candidate for the treatment of bone loss in patients with oxidative stress. However, given the concentration-dependent effects of ROS, we do not recommend prophylactic, prolonged, large-dose use of antioxidants [53]. We have not observed significant changes in the cementum of SOD1(-/-) mice (Figure S4). However, whether the alveolar bone changes of SOD1(-/-) mice will affect the periodontal ligament attached to it and the related mechanisms await further study.

In conclusion, this study showed the dual effects of ROS aggregation on mandibular alveolar bone formation caused by SOD1 deficiency: bone mass (BV/TV, Tb.Th) and expression of osteogenic related proteins (ALP, Runx2, and OPN) increased first and then decreased compared with wild-type mice. This is likely related to the ROS level of the mandibular alveolar bone and the age of the mice. Different concentrations of ROS and even oxidation products (4-HNE) serve as signal molecules, participating in the redox signal network to regulate the normal physiological and pathological state of the body, which is critical in redox biology [54]. We expect that additional exploration in molecular effectors of redox biology in the future will lead to therapies that selectively reduce disease without disturbing healthy tissues.

## Data Availability

The data used to support the findings of this study are available from the corresponding author upon request.

## Supplementary Materials

Supplementary Materials Figure S1: quantitative PCR analysis of DNA of SOD1(-/-) mice, SOD1(+/-) mice, and SOD1(+/+) mice. Figure S2: immunohistochemistry of 4-HNE in alveolar bone under the alveolar crest of the mandibular first molar root bifurcation area of SOD1(-/-) male mice and wild-type male mice and associated quantitative analysis. Data are shown as mean ± SD (*n* = 5). ^∗^*P* < 0.05; ^∗∗^*P* < 0.01. Figure S3: immunohistochemistry of 4-HNE in alveolar bone under the alveolar crest of the mandibular first molar root bifurcation area of SOD1(-/-) male mice and wild-type male mice after adding NAC in diet and associated quantitative analysis. Data are shown as mean ± SD (*n* = 5). ^∗^*P* < 0.05; ^∗∗^*P* < 0.01. Figure S4: HE staining of alveolar bone under the alveolar crest of the mandibular first molar root bifurcation area of SOD1(-/-) male mice and wild-type male mice. D: dentin; PDL: periodontal ligament; C: cementum. Cementum and dentin are distinguished by white dotted lines. Scale bars: 200 *μ*m and 20 *μ*m.
