# Supplementary material for: The Dual Effects of Reactive Oxygen Species on the Mandibular Alveolar Bone Formation in SOD1 Knockout Mice: Promotion or Inhibition
Source: Oxid Med Cell Longev. 2021 Feb 3;2021:8847140. doi: 10.1155/2021/8847140 (PMC7878083; doi:10.1155/2021/8847140)
Supplement: Supplementary Materials — Figure S1: quantitative PCR analysis of DNA of SOD1(-/-) mice, SOD1(+/-) mice, and SOD1(+/+) mice. Figure S2: immunohistochemistry of 4-HNE in alveolar bone under the alveolar crest of the mandibular first molar root bifurcation area of SOD1(-/-) male mice and wild-type male mice and associated quantitative analysis. Data are shown as mean ± SD (n = 5). ∗P < 0.05; ∗∗P < 0.01. Figure S3: immunohistochemistry of 4-HNE in alveolar bone under the alveolar crest of the mandibular first molar root bifurcation area of SOD1(-/-) male mice and wild-type male mice after adding NAC in diet and associated quantitative analysis. Data are shown as mean ± SD (n = 5). ∗P < 0.05; ∗∗P < 0.01. Figure S4: HE staining of alveolar bone under the alveolar crest of the mandibular first molar root bifurcation area of SOD1(-/-) male mice and wild-type male mice. D: dentin; PDL: periodontal ligament; C: cementum. Cementum and dentin are distinguished by white dotted lines. Scale bars: 200 μm and 20 μm. [file 8847140.f1.docx]

Supplementary Material

## Supplementary Figures


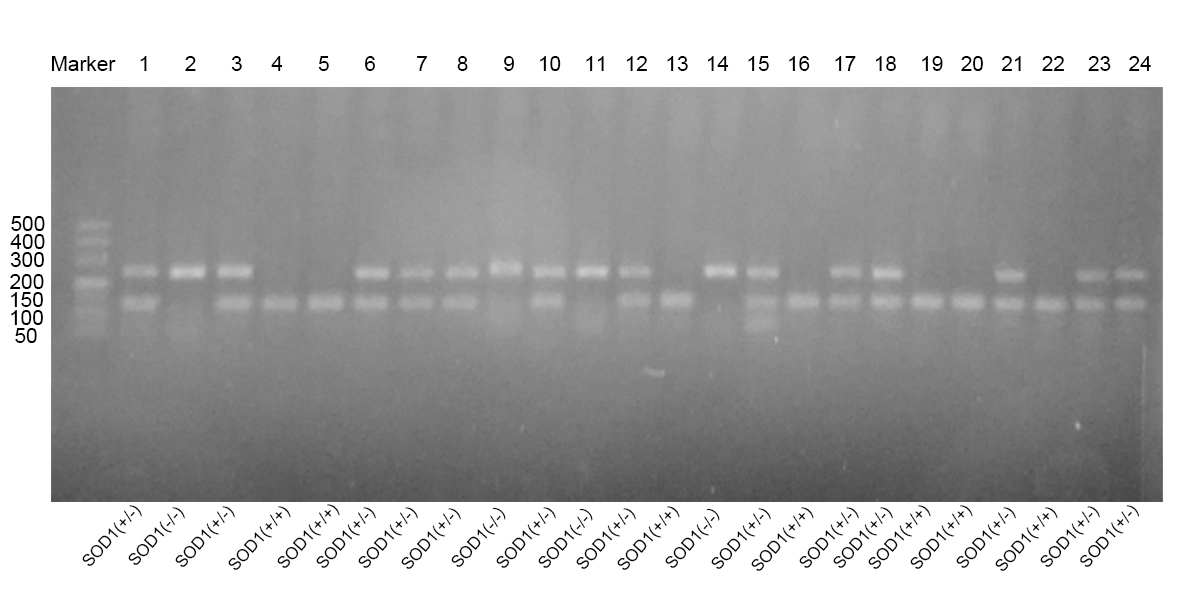
 **Figure S1.** Quantitative PCR analysis of DNA of SOD1(-/-) mice, SOD1(+/-) mice and SOD1(+/+) mice.


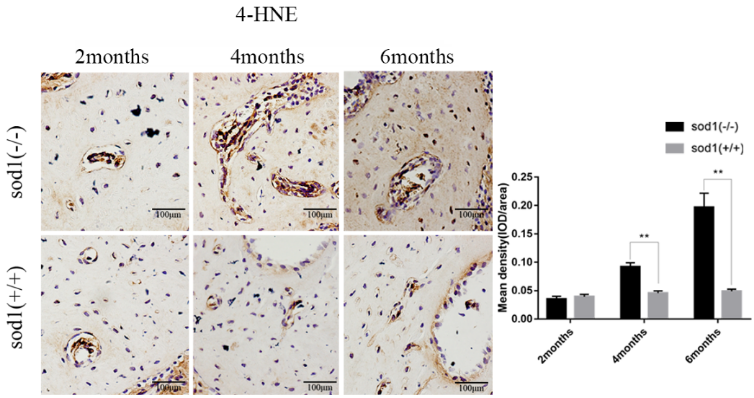


**Figure S2.** Immunohistochemistry of 4-HNE in alveolar bone under the alveolar crest of the mandibular first molar root bifurcation area of SOD1(-/-) male mice and wild-type male mice and associated quantitative analysis. Data are shown as mean ± SD (n=5). *P < 0.05. **P< 0.01.
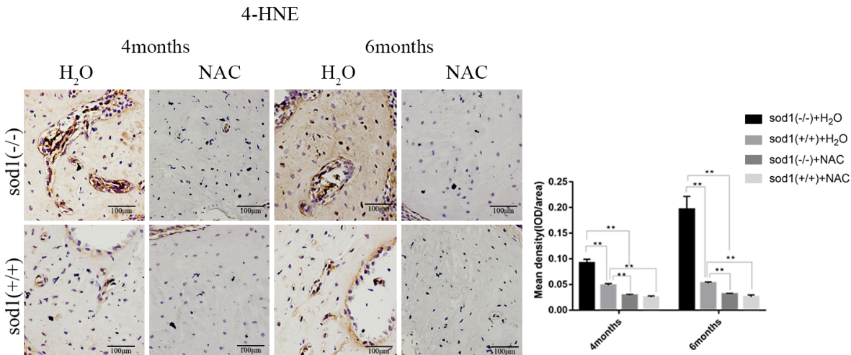


**Figure S3.** Immunohistochemistry of 4-HNE in alveolar bone under the alveolar crest of the mandibular first molar root bifurcation area of SOD1(-/-) male mice and wild-type male mice after adding NAC in diet and associated quantitative analysis. Data are shown as mean ± SD (n=5). *P < 0.05. **P< 0.01.

**
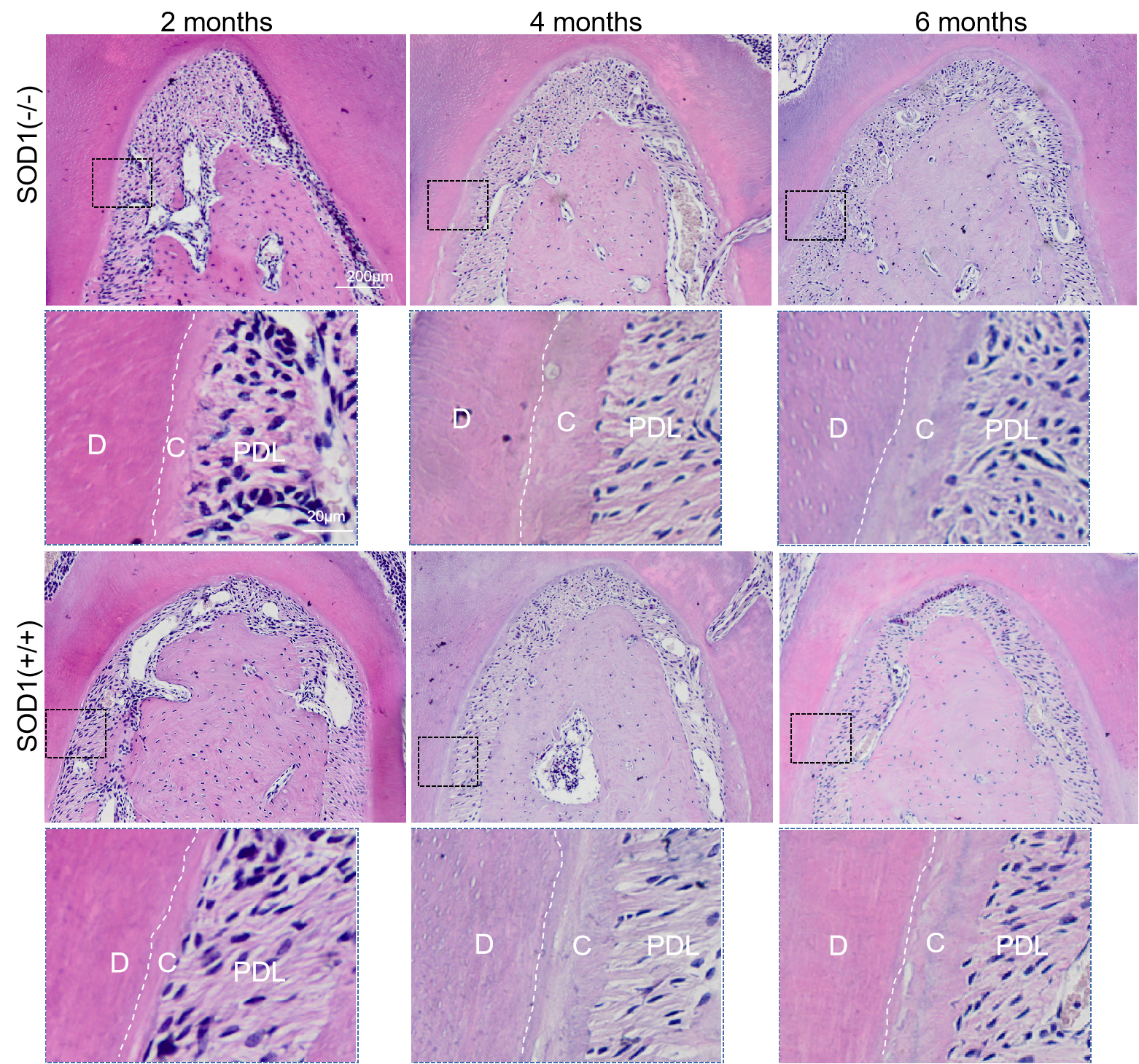
**

**Figure S4. HE staining** of alveolar bone under the alveolar crest of the mandibular first molar root bifurcation area of SOD1(-/-) male mice and wild-type male mice.D, dentin; PDL, periodontal ligament; C, cementum. Cementum and dentin are distinguished by white dotted lines. Scale bars: 200 μm and 20 μm
